# Risk-adaptive management of stage III NSCLC in the perioperative immunotherapy era: a critical synthesis and clinical framework

**DOI:** 10.3389/fonc.2026.1897546

**Published:** 2026-09-08

**Authors:** İbrahim Yıldız, Elif Şenocak Taşçı, Fulya Ağaoğlu

**Affiliations:** 1 Department of Medical Oncology, School of Medicine, Acıbadem Mehmet Ali Aydınlar University, İstanbul, Türkiye; 2 Department of Radiation Oncology, School of Medicine, Acıbadem Mehmet Ali Aydınlar University, İstanbul, Türkiye

**Keywords:** chemo-immunotherapy, circulating tumor DNA, multimodality treatment, perioperative immunotherapy, risk stratification, stage III NSCLC

## Abstract

Stage III non-small cell lung cancer (NSCLC) is a highly heterogeneous disease, and recent phase III trials have demonstrated the efficacy of neoadjuvant and perioperative chemoimmunotherapy in selected patients. However, translating these data into consistent real-world multidisciplinary decision-making remains challenging because anatomical resectability, nodal burden, tumor biology, and treatment response vary substantially. Neoadjuvant nivolumab plus chemotherapy significantly improved pathological complete response and event-free survival (EFS), with a modest but clinically meaningful overall survival (OS) benefit at five years. Perioperative strategies appear to extend these benefits: KEYNOTE-671 demonstrated a significant OS advantage, while AEGEAN and CheckMate 77T showed consistent EFS improvements, with OS data still maturing. However, cross-trial comparisons are inherently limited, and the incremental contribution of the adjuvant immunotherapy phase remains unclear. Exploratory subgroup analyses suggest potentially greater benefit in N2 disease, although prospective validation is required. For tumors harboring actionable oncogenic alterations, upfront resection followed by adjuvant targeted therapy remains the preferred strategy, whereas definitive chemoradiotherapy followed by consolidation durvalumab remains standard of care for unresectable stage III disease. Management of stage III NSCLC should be individualized through multidisciplinary assessment. Major uncertainties persist regarding the comparative efficacy of perioperative versus neoadjuvant-only strategies, the contribution of adjuvant immunotherapy, and the role of biomarker-guided treatment adaptation. This clinical framework provides structured guidance to support treatment selection across the heterogeneous spectrum of stage III disease, while recognizing that prospective validation of risk-adapted strategies remains essential.

## Introduction

Stage III non-small cell lung cancer (NSCLC) remains one of the most heterogeneous and clinically challenging entities in thoracic oncology. It encompasses a broad clinical spectrum, ranging from clearly resectable tumors with limited mediastinal nodal involvement to anatomically complex disease with extensive multistation N2 involvement, for which nonsurgical strategies are often favored (1, 2). This anatomical and biological diversity limits the applicability of uniform treatment algorithms and necessitates individualized, multidisciplinary decision-making.

The subdivision of N2 disease into single-station (N2a) and multistation (N2b) categories reflects the increasing recognition of prognostic and therapeutic heterogeneity within stage III disease, with important implications for treatment selection and intensity (3, 4). Patients with limited N2a disease may achieve favorable outcomes with surgery-based multimodality strategies, whereas those with bulky or multistation mediastinal involvement often require intensified systemic therapy or definitive chemoradiotherapy to achieve durable disease control.

The integration of immune checkpoint inhibitors (ICIs) into multimodality treatment paradigms has substantially reshaped stage III NSCLC management. Multiple phase III trials have demonstrated that neoadjuvant and perioperative chemoimmunotherapy significantly improve pathological response rates and event-free survival (EFS), with overall survival (OS) benefit reported in selected settings; the single-arm phase II NADIM trial has also provided supportive early evidence for this approach (5–9). For unresectable stage III disease, consolidation immunotherapy following definitive concurrent chemoradiotherapy has become the standard of care based on the PACIFIC trial (10, 11).

Despite these advances, a substantial gap persists between clinical trial evidence and real-world multidisciplinary decision-making. Although pivotal trials have established the efficacy of neoadjuvant and perioperative immunotherapy, they do not provide a unified operational framework for selecting treatment according to anatomical resectability, nodal burden, tumor biology, and dynamic response to induction therapy. Current guidelines acknowledge these complexities but offer limited guidance on tailoring treatment intensity or selecting between neoadjuvant-only and full perioperative strategies, resulting in considerable institutional variability (2–12).

**Figure 1:**
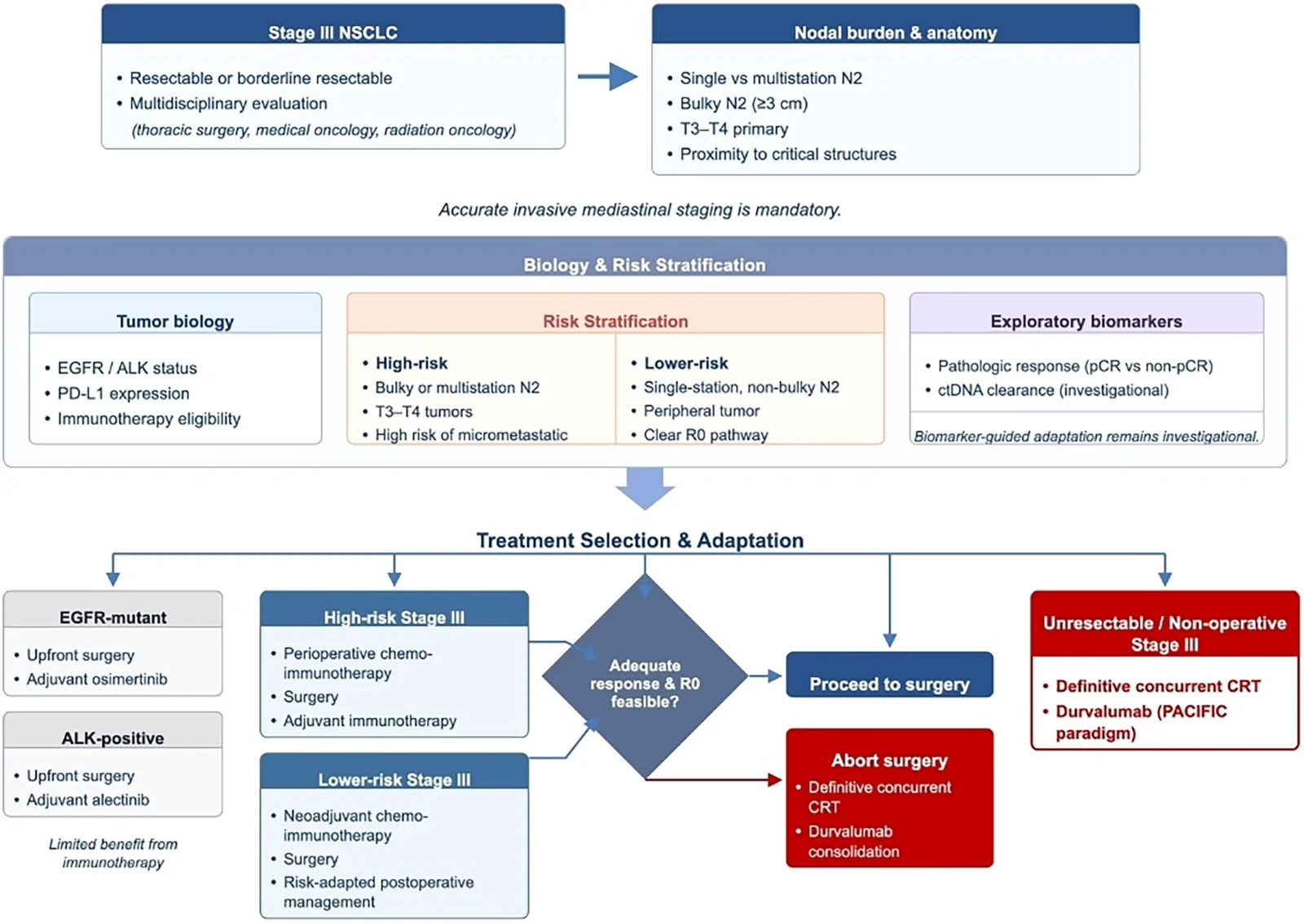
Risk-adaptive treatment algorithm for stage III NSCLC in the perioperative immunotherapy era. The proposed framework integrates anatomical resectability (based on EBUS/mediastinoscopy findings and cross-sectional imaging), nodal burden (single-station N2a vs multistation N2b), tumor biology (EGFR/ALK alterations and PD-L1 expression), and patient functional status to inform treatment strategy. Blue boxes indicate diagnostic and staging steps; yellow boxes represent risk stratification; red denotes surgical management; orange indicates the chemoradiotherapy pathway; and purple highlights biomarker evaluation. Transition points for inadequate response to induction therapy reflect expert consensus pending prospective validation. *pCR, pathologic complete response; ctDNA, circulating tumor DNA; ECOG, Eastern Cooperative Oncology Group; CRT, chemoradiotherapy; EGFR, epidermal growth factor receptor; ALK, anaplastic lymphoma kinase; PD-L1, programmed death-ligand 1; EBUS, endobronchial ultrasound; IO, immunotherapy*.

In this review, we propose a risk-adaptive, biology-driven framework that integrates anatomical complexity, tumor biology, and dynamic response assessment to support individualized multidisciplinary decision-making in stage III NSCLC (**Figure 1**). By synthesizing contemporary phase III data with emerging biomarker evidence and practical surgical and radiotherapeutic considerations, this framework aims to provide structured guidance for treatment selection across the heterogeneous spectrum of resectable and borderline-resectable stage III disease. Rather than replacing current guidelines, the proposed framework is intended to operationalize their principles by integrating dynamic response assessment with anatomical and biological risk. It also proposes explicit transition points for patients with inadequate response to neoadjuvant therapy, a decision area that current recommendations (NCCN, IASLC, ASCO, and ESMO) largely leave to clinician discretion.

## Literature search and methodology

In accordance with the Scale for the Assessment of Narrative Review Articles (SANRA), we searched PubMed/MEDLINE, Embase, and the Cochrane Library through May 2026 using combinations of the terms “stage III NSCLC,” “neoadjuvant,” “perioperative,” “immunotherapy,” “chemoimmunotherapy,” “ctDNA,” and “chemoradiotherapy,” supplemented by hand-searching of ASCO, ESMO, and WCLC proceedings and reference lists. Priority was given to phase III randomized trials and high-quality systematic reviews; congress abstracts were included where they represented the most current data but were explicitly flagged as preliminary. Included publications were critically appraised with explicit weighting of peer-reviewed versus congress-presented evidence and grading of exploratory subgroup analyses.

## Diagnosis, staging and molecular profiling: foundational principles

Accurate staging is the cornerstone of therapeutic decision-making in stage III NSCLC, as treatment strategies differ fundamentally according to tumor extent, mediastinal nodal involvement, and patient operability (1, 2). Integrated imaging with contrast-enhanced computed tomography and fluorodeoxyglucose positron emission tomography/computed tomography remains essential for treatment planning.

Given the clinically meaningful incidence of occult brain metastases in locally advanced NSCLC—particularly in patients with adenocarcinoma histology or extensive nodal disease—baseline contrast-enhanced brain magnetic resonance imaging is recommended for all patients with stage III disease (13). Identification of intracranial disease reclassifies patients to stage IV and redirects management.

Endobronchial ultrasound-guided transbronchial needle aspiration, with or without complementary endoscopic ultrasound-guided sampling, is the preferred minimally invasive approach for mediastinal nodal staging, offering high diagnostic accuracy with low procedural morbidity (14, 15). When minimally invasive staging yields negative or inconclusive results despite high clinical suspicion—such as in the presence of bulky nodes, central tumors, or inaccessible stations—surgical staging with mediastinoscopy remains warranted to ensure accurate treatment allocation (15, 16).

Comprehensive molecular profiling is vital, as actionable oncogenic driver alterations—including EGFR mutations, ALK rearrangements, and other targetable events—substantially influence perioperative strategy selection and anticipated immunotherapy benefit (17–20). Patients with sensitizing EGFR mutations or ALK rearrangements generally derive limited benefit from ICIs and may be more appropriately managed with surgery followed by adjuvant targeted therapy when disease is resectable.

For the purposes of this framework, resectable disease denotes tumors in which an R0 resection is achievable with acceptable morbidity; potentially (borderline) resectable disease denotes tumors in which R0 is uncertain and contingent on induction response and multidisciplinary judgment; and unresectable disease denotes tumors in which R0 cannot be achieved (e.g., bulky multistation N2/N3 involvement or T4 invasion of unresectable structures). This resectability determination should be made by a multidisciplinary team.

## Evidence for neoadjuvant-only chemoimmunotherapy

As summarized in **Table 1**, the phase III CheckMate 816 trial provided the first randomized evidence supporting the addition of an ICI to neoadjuvant chemotherapy in resectable NSCLC (5). Neoadjuvant nivolumab plus chemotherapy significantly improved pathological complete response (pCR) rates (24.0% vs 2.2%) and major pathological response rates compared with chemotherapy alone, and this was accompanied by a clinically meaningful improvement in EFS (HR 0.63; 97.38% CI 0.43–0.91) (5).

**Table 1:** Key clinical trials in stage III NSCLC: pathological response, survival outcomes, and biomarker correlates.

Trial [ref]	Population	Treatment	Pathological (pCR/MPR)	Survival (EFS/OS)	PD-L1 subgroup	ctDNA/biomarker	Clinical implication
CheckMate 816 (5, 21)	Resectable IB–IIIA (≈65% IIIA)	Neoadjuvant nivolumab + chemo	pCR 24.0% vs 2.2%; MPR 36.9% vs 11.0%	EFS HR 0.63; 5-yr OS 65% vs 55%, HR 0.72 (P = 0.048)	Benefit across all PD-L1 strata (greater if ≥1%)	pCR linked to excellent long-term OS (>85% at 5 yr); ctDNA clearance favorable	Establishes neoadjuvant-only chemo-IO with proven OS benefit
CheckMate 77T (7, 28, 30)	Resectable IIA–IIIB (Stage III 64%; N2 ~40%)	Perioperative nivolumab + chemo	Overall pCR 25.3% vs 4.7%; N2 pCR 22.0% vs 5.6%; multistation N2 pCR 29.0% vs 2.7%	30-mo EFS HR 0.61; N2 1-yr 70% vs 45% (HR 0.46); multistation N2 1-yr 71% vs 46% (HR 0.43); 30-mo OS HR 0.85 (immature)	Benefit across all strata; HR 0.52 (≥1%) vs 0.73 (<1%)	Presurgical ctDNA clearance correlates with EFS; N0 downstaging 57% vs 44%	May improve N2 outcomes (exploratory); prospective N2-enriched validation warranted
KEYNOTE-671 (8, 26)	Resectable II–IIIB (Stage III 70%; N2 ~43%)	Perioperative pembrolizumab + chemo	pCR 18.1% vs 4.0%; MPR 30.2% vs 10.7%	5-yr EFS 49.9% vs 26.5% (HR 0.58); 5-yr OS 64.6% vs 53.6% (HR 0.74)	EFS benefit regardless of PD-L1 (numerically greater ≥50%)	Non-pCR = major residual-risk group; ctDNA-guided de-escalation under study	Supports perioperative intensification for high-risk stage III
AEGEAN (27, 29)	Resectable II–IIIB (Stage III 67%; N2 ~50%)	Perioperative durvalumab + chemo	pCR 17.2% vs 4.3%; MPR 33.3% vs 12.1%	EFS HR 0.68; OS immature (mOS NR vs 53.2 mo; HR 0.89)	Strongest benefit in N2; also observed in the PD-L1-low subgroup	Residual disease common; prolonged immune control likely relevant	Reinforces perioperative approach in bulky/multistation N2
PACIFIC (10, 11)	Unresectable stage III	cCRT → durvalumab consolidation	N/A	PFS HR 0.52; 5-yr OS 42.9% vs 33.4% (HR 0.72)	Benefit independent of PD-L1	ctDNA clearance after CRT linked to durable control (exploratory)	Standard of care for unresectable stage III
PACIFIC-2 (45)	Unresectable stage III	Concurrent durvalumab + cCRT	N/A	PFS HR 0.85 (P = 0.247, NS); OS HR 1.03 (P = 0.823, NS)	No PD-L1-specific advantage	No added biological advantage demonstrated	Sequential (not concurrent) IO integration preferred
NeoADAURA (52)	EGFR-mutant resectable II–IIIB	Neoadjuvant osimertinib ± chemo	MPR ≈25–26% vs ≈2%	EFS immature; OS immature	PD-L1 not predictive in EGFR+	ctDNA suppression promising but exploratory	Emerging neoadjuvant option; phase III ongoing
ALNEO (Ph II) (53)	ALK-positive stage III	Neoadjuvant alectinib → surgery → adjuvant alectinib	MPR ≈40%; pCR ≈10–15%	Survival data immature	PD-L1 not relevant in ALK+	Deep molecular responses support TKI-centered paradigm	Supports biology-driven TKI strategy in ALK+

pCR, pathological complete response; MPR, major pathological response; EFS, event-free survival; OS, overall survival; HR, hazard ratio; CI, confidence interval; PD-L1, programmed death-ligand 1; ctDNA, circulating tumor DNA; cCRT, concurrent chemoradiotherapy; PFS, progression-free survival; TKI, tyrosine kinase inhibitor; N/A, not applicable; NS, not significant; IO, immunotherapy; N2, ipsilateral mediastinal/subcarinal lymph node metastases; N0, node-negative disease.

Several citations correspond to data presented at international congresses (ESMO 2025, ASCO 2025, WCLC 2024) and represent preliminary findings pending full peer-reviewed publication. N2 subgroup analyses are exploratory and were not pre-specified primary endpoints; these findings should be interpreted as hypothesis-generating and require prospective confirmation in N2-enriched trials.

With extended follow-up, five-year OS was 65% versus 55% (HR 0.72; 95% CI 0.52–0.99), supporting the durability and clinical relevance of the early pathological response benefit (21). Benefit was observed across clinically relevant subgroups, including PD-L1 expression, disease stage, and nodal burden (5, 6, 21). Although effect sizes were numerically greater in PD-L1–positive tumors, meaningful benefit observed in PD-L1–negative disease supports broad applicability and reinforces that PD-L1 expression alone should not serve as an exclusion criterion.

The phase II NADIM trial further provided additional supportive evidence for the feasibility and efficacy of neoadjuvant chemo-immunotherapy in stage IIIA NSCLC, demonstrating high pathological response rates and encouraging long-term survival outcomes (9).

Within our framework (**Figure 1**), neoadjuvant-only chemoimmunotherapy is an evidence-supported treatment option; its restriction to carefully selected lower-risk patients—those with a high likelihood of R0 resection, including single-station N2a disease, limited mediastinal tumor burden, and favorable tumor biology—reflects our expert opinion rather than randomized evidence, as CheckMate 816 was not designed to test such a risk-stratified selection rule. A recent meta-analysis comparing immunotherapy-based neoadjuvant or perioperative strategies with chemotherapy-alone approaches further supports immunotherapy integration prior to surgery (22).

Upfront surgery without induction therapy may remain reasonable in highly selected scenarios, including limited nodal disease, oncogene-driven tumors for which adjuvant targeted therapy is planned, contraindications to ICIs, or cases in which multidisciplinary consensus anticipates minimal incremental benefit from neoadjuvant treatment (2, 22, 23).

## Adjuvant-only immunotherapy

Adjuvant-only immunotherapy represents a mechanistically distinct postoperative strategy. IMpower010 (adjuvant atezolizumab) and PEARLS/KEYNOTE-091 (adjuvant pembrolizumab) both demonstrated disease-free survival (DFS) benefit in resected NSCLC and subsequently received regulatory approval (24, 25). However, the magnitude and distribution of benefit were not uniform across clinically relevant subgroups. In IMpower010, the DFS benefit was most pronounced in PD-L1–positive stage II–IIIA disease and did not meet the significance boundary in the overall intention-to-treat (stage IB–IIIA) population, whereas PEARLS/KEYNOTE-091 demonstrated a significant DFS benefit in the overall population (HR 0.76; 95% CI 0.63–0.91) but not in the PD-L1 TPS ≥50% subgroup (HR 0.82; 95% CI 0.57–1.18). In contrast to neoadjuvant and perioperative approaches, adjuvant-only delivery forgoes the opportunity for *in situ* immune priming against intact tumor antigen and for early pathological response assessment. The absence of subsequent trials pursuing adjuvant-only chemoimmunotherapy, together with the pronounced shift of the field toward neoadjuvant and perioperative designs, further underscores the limitations of a purely postoperative strategy.

## Evidence for perioperative chemoimmunotherapy

Perioperative immunotherapy has extended the neoadjuvant treatment paradigm by incorporating checkpoint inhibition both before and after surgery. Compared with adjuvant-only, perioperative strategies offer potential biological advantages by delivering systemic therapy earlier, promoting immune priming in the presence of intact tumor antigen, and potentially maintaining postoperative immune pressure against residual micrometastatic disease. However, neoadjuvant and perioperative strategies may provide additional advantages through earlier systemic disease control and tumor antigen-driven immune activation, although direct clinical confirmation that these mechanisms mediate the observed survival benefits remains lacking. In this context, three phase III trials—KEYNOTE-671, AEGEAN, and CheckMate 77T—demonstrated consistent improvements in pathological response and EFS across resectable stage II–III NSCLC (**Table 1**) (26–28).

KEYNOTE-671 evaluated perioperative pembrolizumab in 797 patients with resectable stage II, IIIA, or IIIB (N2) NSCLC (8). Pembrolizumab significantly improved EFS (HR 0.58; 95% CI 0.46–0.72) and pCR rates (18.1% vs 4.0%). At the five-year follow-up presented at ESMO 2025, perioperative pembrolizumab continued to show clinically meaningful benefit, with OS rates of 64.6% versus 53.6% (HR 0.74; 95% CI 0.59–0.92) and EFS rates of 49.9% versus 26.5%; however, these data should be interpreted in the context of a conference presentation pending full peer-reviewed publication (26).

AEGEAN tested perioperative durvalumab in 802 patients with resectable stage II–IIIB NSCLC (27). Durvalumab improved pCR (17.2% vs 4.3%) and EFS (HR 0.68; 95% CI 0.53–0.88), with subgroup analyses suggesting more pronounced benefit among patients with stage III and N2 disease. Updated survival data presented at WCLC 2024 showed continued EFS benefit and a favorable, but not yet definitive, OS trend (median OS not reached vs 53.2 months; HR 0.89; 95% CI 0.70–1.14), supporting continued follow-up before firm conclusions regarding OS can be drawn (29).

CheckMate 77T evaluated perioperative nivolumab in 461 patients with resectable stage IIA–IIIB NSCLC, demonstrating significant EFS improvement (HR 0.58; 97.36% CI 0.42–0.81) with higher pCR and major pathological response rates (7). Updated survival and biomarker analyses, presented at ASCO 2025, have shown sustained EFS benefit, whereas OS data remain immature (30).

Additional phase III evidence from Asia further supports the activity of perioperative immunotherapy in resectable stage III NSCLC. In the NEOTORCH trial, perioperative toripalimab plus chemotherapy significantly improved event-free survival compared with chemotherapy alone (HR 0.40; 95% CI 0.28–0.57), with higher pCR rates (24.8% vs 6.0%) in a predominantly East Asian population (31).

Across perioperative trials, adjuvant immunotherapy was administered for the full protocol-specified duration regardless of pathological response (7, 8, 27). Exploratory analyses from CheckMate 77T revealed clinically meaningful benefit in N2 disease, with 1-year EFS of 70% versus 45% (HR 0.46; 95% CI 0.30–0.70) and pCR of 22.0% versus 5.6% (28). In multistation N2 disease, perioperative nivolumab achieved 1-year EFS of 71% versus 46% (HR 0.43; 95% CI 0.21–0.88), pCR of 29.0% versus 2.7%, with nodal downstaging to N0 in 57% versus 44%. Although these findings suggest that perioperative immunotherapy may be especially valuable in patients with higher-risk nodal disease, they remain exploratory and should be confirmed in prospectively designed, N2-enriched studies.

Whether the adjuvant immunotherapy contributes meaningfully beyond neoadjuvant chemoimmunotherapy alone remains a critical unresolved question, as no randomized trial has directly compared neoadjuvant-only versus full perioperative strategies. The benefits observed with perioperative regimens may be driven largely by the neoadjuvant component, while the incremental value of postoperative continuation cannot be reliably quantified from current trial designs. This uncertainty is particularly relevant for patients achieving pCR, in whom continued adjuvant immunotherapy entails cumulative toxicity, financial burden, and treatment burden despite the lack of clearly established incremental benefit in this subgroup. Recent meta-analyses comparing perioperative with neoadjuvant-only strategies provide useful indirect evidence but cannot substitute for a head-to-head randomized comparison (22, 23).

A practical consideration is that 15–25% of patients in major neoadjuvant trials did not ultimately undergo resection due to progression, toxicity, or clinical deterioration, highlighting the importance of early response assessment and timely multidisciplinary reassessment (5–8, 21, 27, 30).

Within our proposed expert-opinion framework (**Figure 1**), perioperative chemo-immunotherapy may be preferentially considered for patients with higher-risk resectable or borderline-resectable stage III disease, including bulky or multistation N2 disease and anatomically complex presentations with elevated recurrence risk. This allocation is informed by, but not directly established by, the exploratory subgroup analyses discussed above.

## Pathological and molecular response assessment: pCR and ctDNA

Patients achieving pCR following neoadjuvant chemoimmunotherapy have consistently shown markedly favorable long-term outcomes, supporting pCR as a clinically meaningful prognostic marker in resectable NSCLC. In the final five-year analysis of CheckMate 816, patients who achieved pCR after nivolumab plus chemotherapy had particularly favorable survival, with five-year OS reported at 95% compared with 56% among those without pCR (21). However, reliable preoperative pCR identification using conventional imaging remains challenging, as radiographic response does not reliably exclude residual microscopic disease.

Circulating tumor DNA (ctDNA) has emerged as a promising minimally invasive biomarker for molecular response assessment and postoperative risk stratification. Early translational studies demonstrated that postoperative ctDNA detection reflects molecular residual disease and is strongly associated with early recurrence, even among patients achieving pCR (32, 33). These findings suggest that molecular response may provide complementary biological information beyond radiographic and pathological assessment by identifying residual disease that is not detectable using conventional methods.

Prospective studies have further demonstrated that perioperative ctDNA clearance is associated with significantly improved disease-free survival (DFS) and OS, whereas persistent detectability identifies patients at high risk of recurrence and may define a population for whom treatment intensification should be investigated (34, 35).

Across major trials, approximately 70–80% of patients do not achieve pCR (5–8, 30). Exploratory biomarker analyses from CheckMate 77T (presented at ASCO 2025) suggest that ctDNA clearance before surgery is associated with improved EFS irrespective of pathological response category, raising the possibility that molecular response assessment could complement pathological response evaluation (30). However, these findings remain hypothesis-generating and require prospective validation.

Current perioperative trial protocols mandate adjuvant immunotherapy continuation regardless of pathological response or ctDNA status (7, 8, 30). No prospective randomized data support routine escalation or de-escalation based on pCR or ctDNA kinetics outside clinical trials, and premature modification of treatment intensity may risk undertreatment in biologically aggressive disease.

Beyond clinical validation, the routine implementation of ctDNA in stage III NSCLC faces substantive methodological barriers. Analytical validity and inter-assay variability remain major challenges, with commercial platforms differing in limits of detection, mutation panel coverage, and bioinformatics pipelines, thereby complicating cross-study comparison and clinical standardization. False-negative results are particularly concerning in low-shedding tumors and certain non-adenocarcinoma histologies, while uncertainties regarding the optimal timing of postoperative blood draws further limit clinical decision-making. Until tumor-informed and tumor-naive assays are prospectively standardized and validated for treatment-modifying decisions, ctDNA-guided escalation or de-escalation should remain confined to investigational settings (36, 37).

In summary, pCR and ctDNA dynamics represent complementary biomarkers with substantial prognostic value. While promising for enabling future response-adaptive strategies, their role in guiding routine therapy modification remains investigational and requires prospective validation.

## Role of radiotherapy in the immunotherapy era

Radiotherapy continues to play a critical complementary role in stage III NSCLC management. Although contemporary chemoimmunotherapy regimens achieve substantially higher pathological response rates than historical chemoradiotherapy approaches, radiotherapy remains essential when anatomical factors limit complete resection with systemic therapy alone (5–8, 38–40).

Treatment selection should be guided by whether anatomical complexity or adverse tumor biology represents the dominant barrier to curative-intent surgery. Tumors involving critical mediastinal structures—including major airways, pulmonary arteries, or superior vena cava—may derive greater locoregional benefit from neoadjuvant chemoradiotherapy to facilitate margin-negative resection (41–43). On the other hand, when extensive nodal burden or concern for micrometastatic disease predominates, chemoimmunotherapy may be favored to maximize early systemic disease control.

Patients with inadequate response to induction therapy should undergo prompt multidisciplinary reassessment, as high-risk surgery in this setting is unlikely to achieve R0 resection and may expose patients to substantial morbidity without clear oncologic benefit (41–43). In such cases, transition to definitive chemoradiotherapy, with consolidation immunotherapy when appropriate, should be considered rather than persisting with a surgical strategy of low curative probability.

For unresectable stage III NSCLC, the PACIFIC trial established consolidation durvalumab as standard of care, with five-year OS of 42.9% versus 33.4% (HR 0.72; 95% CI 0.59–0.89) (10, 11). Real-world data from PACIFIC-R have further supported the generalizability of the PACIFIC regimen in routine practice (44).

The phase III PACIFIC-2 trial, which evaluated concurrent durvalumab with chemoradiotherapy followed by consolidation durvalumab, failed to improve PFS (HR 0.85; 95% CI 0.66–1.10; P = 0.247) or OS (HR 1.03; 95% CI 0.75–1.41; P = 0.823), reinforcing sequential rather than concurrent integration of immunotherapy with radiotherapy in unresectable stage III disease (45). The optimal integration of radiotherapy after immune checkpoint blockade nonetheless remains debated, particularly regarding the risk of radiation pneumonitis when radiotherapy follows immunotherapy; careful dosimetric and temporal planning is therefore warranted.

Importantly, the consolidation strategy after definitive chemoradiotherapy is increasingly shaped by tumor biology. In EGFR-mutant unresectable stage III NSCLC, the phase III LAURA trial demonstrated that consolidation osimertinib significantly improved progression-free survival after definitive chemoradiotherapy (HR 0.16; 95% CI 0.10–0.24), establishing a biology-driven consolidation approach for this molecular subgroup (46). This finding further underscores the need for comprehensive molecular profiling before definitive treatment decisions are finalized, even in stage III disease.

## Surgical considerations in the immunotherapy era

Neoadjuvant chemoimmunotherapy has not appeared to compromise surgical feasibility or result in increased perioperative morbidity when patients were appropriately selected and surgery was performed within 4–6 weeks after induction therapy (5–8). When technically feasible, lung-preserving surgical strategies—including lobectomy rather than pneumonectomy, sleeve lobectomy, and bronchoplastic or angioplastic resections—should be prioritized to avoid pneumonectomy without compromising oncologic outcomes (42, 47).

Chemoimmunotherapy may facilitate tumor and nodal downstaging, thereby increasing the feasibility of R0 resection and parenchymal preservation in selected patients (5–8). Across perioperative trials, R0 resection rates among patients undergoing definitive surgery remained high (approximately 89–92% in the overall populations of CheckMate 77T and KEYNOTE-671), while pneumonectomy rates remained acceptable relative to historical surgical series (7, 8, 30).

Pneumonectomy after chemoimmunotherapy remains controversial and should be reserved for carefully selected cases in experienced centers (48–50). Current evidence supports pneumonectomy only when R0 resection cannot be achieved with parenchymal-sparing techniques, with decisions individualized according to performance status, pulmonary function reserve, comorbidity burden, and institutional surgical experience (51). In the N2 subgroup specifically from CheckMate 77T, pneumonectomy rates were notably lower with nivolumab (1%) than with placebo (14%), while R0 rates remained comparable (86% vs 86%), further supporting that perioperative immunotherapy may facilitate parenchymal preservation (28).

For tumors harboring actionable oncogenic driver alterations, including sensitizing EGFR mutations or ALK rearrangements, the role of immunotherapy-based neoadjuvant or perioperative treatment is less certain. When complete resection is achievable, upfront surgery followed by guideline-concordant adjuvant targeted therapy is frequently preferred (17–20). Phase III trials have demonstrated significant DFS and OS benefit with adjuvant osimertinib in a EGFR-mutant and with alectinib in ALK-positive NSCLC (19, 20). Neoadjuvant targeted strategies, including NeoADAURA and the phase II ALNEO trial (presented at ASCO 2025), are promising but remain non-standard outside clinical trials pending mature survival data and broader clinical validation (52, 53).

Immune-related pneumonitis, though infrequent, represents a critical determinant of surgical timing and feasibility. Surgery should generally be deferred until pneumonitis has clinically improved to grade ≤1, radiographic findings are stable, and systemic corticosteroids have been appropriately tapered (54, 55). These decisions require multidisciplinary assessment, particularly when suspected pneumonitis overlaps radiographically with post-obstructive changes, infection, or radiation-related lung injury.

Beyond pneumonitis, other immune-related adverse events—including endocrinopathies (hypophysitis, thyroid dysfunction), hepatitis, colitis, dermatologic toxicity, and the rare but potentially fatal myocarditis and neurologic syndromes—may also affect surgical planning. Active grade ≥2 events, or the requirement for immunosuppressive corticosteroid doses, generally warrant deferral of surgery until clinical resolution and appropriate taper, in accordance with ASCO and ESMO management guidelines (54, 55). Endocrine events in particular may necessitate perioperative hormone replacement and stress-dose corticosteroid coverage.

Beyond perioperative safety, surgical quality remains essential in the immunotherapy era. Systematic mediastinal lymph node dissection should therefore be performed at the time of resection, even in patients with apparent radiographic or metabolic nodal response, because residual microscopic disease may persist despite imaging response (15, 51).

## Management of inadequate response to neoadjuvant therapy

Radiographic progression, persistent bulky multistation N2 involvement, metabolic non-response on reassessment imaging, or clinical deterioration during neoadjuvant chemoimmunotherapy should prompt immediate multidisciplinary reassessment (51). Proceeding with surgery in patients with an inadequate response is unlikely to achieve durable disease control when R0 resection is no longer feasible and may expose patients to substantial perioperative morbidity without meaningful oncologic benefit.

In the absence of distant progression, timely transition to definitive concurrent chemoradiotherapy should be considered, with consolidation immunotherapy reserved for eligible patients without prohibitive toxicity or disease progression after chemoradiotherapy. This approach is broadly informed by the established role of definitive chemoradiotherapy followed by consolidation durvalumab in unresectable stage III NSCLC, as demonstrated in the PACIFIC trial (10, 11). Importantly, however, the PACIFIC evidence base derives from patients with *de novo* unresectable disease treated with definitive chemoradiotherapy, rather than from patients who progressed or deteriorated after induction chemoimmunotherapy; it therefore cannot be directly extrapolated to the post-neoadjuvant-failure setting, and some of these patients may be too deconditioned to tolerate definitive chemoradiotherapy.

Early identification of treatment failure and avoidance of futile high-risk surgery are critical decision points incorporated into our algorithm (**Figure 1**). Regular multidisciplinary tumor board review after induction therapy is essential to ensure timely treatment adaptation and preserve curative intent whenever possible.

## Critical appraisal of current evidence

Several substantive uncertainties limit confident generalization of trial findings to routine practice. First, no head-to-head randomized comparison exists between neoadjuvant-only, as evaluated in CheckMate 816, and full perioperative strategies, as evaluated in KEYNOTE-671, AEGEAN, and CheckMate 77T. Consequently, the incremental contribution of postoperative immunotherapy remains indirectly inferred from cross-trial comparisons rather than directly established (22, 23).

Second, although subgroup analyses from perioperative trials suggest clinically meaningful benefit in patients with N2 disease, these analyses were exploratory and not powered as primary treatment-defining endpoints. The apparent magnitude of benefit in this clinically relevant population should therefore be regarded as hypothesis-generating and requires confirmation in prospectively designed, N2-enriched trials (28).

Third, although pCR and ctDNA dynamics show promise as response biomarkers, current evidence does not support treatment escalation or de-escalation based on these markers outside investigational settings, since premature modification of treatment intensity could risk undertreatment in biologically aggressive disease (30, 32–35).

Finally, pivotal trials enrolled predominantly ECOG 0–1 patients, leaving uncertain the applicability of perioperative regimens to frail patients and those with substantial comorbidity (5–8). The cumulative toxicity, financial burden, and patient inconvenience associated with prolonged adjuvant immunotherapy remain inadequately quantified relative to the uncertain magnitude of incremental benefit, particularly in patients achieving pCR. Until randomized data demonstrate otherwise, completion of the protocol-specified adjuvant phase remains the evidence-based default, whereas response-adapted de-escalation—particularly in patients achieving pCR with concurrent ctDNA clearance—should be regarded as a high-priority hypothesis for prospective evaluation rather than current practice.

## Proposed risk-adaptive treatment selection framework

As illustrated in **Figure 1**, optimal management of stage III NSCLC requires structured triage between surgery-based curative-intent pathways and definitive nonsurgical strategies through integrated assessment of anatomical resectability, nodal burden, tumor biology, and patient functional status. We propose a scenario-based clinical decision framework that maps common multidisciplinary decision scenarios to suggested treatment approaches with supporting evidence (**Table 2**). Throughout this framework, we distinguish evidence-based recommendations—those directly supported by phase III randomized trials—from expert-opinion recommendations, which rest on cross-trial comparison, exploratory subgroup analyses, or multidisciplinary consensus in the absence of definitive randomized data.

**Table 2:** Scenario-based clinical decision framework for stage III NSCLC in the perioperative immunotherapy era.

Clinical scenario	Key risk feature	Suggested approach	Rationale and key evidence
Single-station N2a (non-bulky)	Low nodal burden; peripheral tumor; clear R0 pathway	Neoadjuvant chemo-IO → surgery ± adjuvant IO	High probability of R0 resection with limited systemic risk. CheckMate 816 (5, 21): pCR 24% vs 2.2%, 5-yr OS 65% vs 55% (HR 0.72). Neoadjuvant-only may be adequate in favorable-biology tumors. (Evidence-based)
Multistation N2b	High nodal burden; elevated micrometastatic risk	Perioperative chemo-IO (full neoadjuvant + adjuvant course)	Exploratory CheckMate 77T N2 subgroup: EFS HR 0.43; pCR 29% vs 2.7%; N0 downstaging 57% vs 44% (28). KEYNOTE-671 5-yr OS HR 0.74 (26). Targets occult systemic disease. (Exploratory/expert opinion)
Bulky N2 (≥3 cm) or T4 with mediastinal invasion	Anatomical complexity; high R1/R2 risk; proximity to critical structures	Perioperative chemo-IO or neoadjuvant CRT → surgery	Selection guided by dominant barrier to resection: anatomical complexity favors CRT for local control; systemic risk favors chemo-IO. STS expert consensus supports individualized assessment (41). (Expert opinion)
EGFR-mutant or ALK-positive	Oncogenic driver alteration; limited IO benefit	Upfront surgery → adjuvant targeted therapy (osimertinib/alectinib)	Adjuvant osimertinib DFS/OS benefit (17, 19); adjuvant alectinib (20). Limited ICI efficacy in oncogene-driven tumors (18). Neoadjuvant TKI strategies remain investigational (52, 53). (Evidence-based for adjuvant TKI)
Inadequate response to neoadjuvant therapy	Radiographic progression; persistent bulky disease; lack of metabolic response	Abort surgery → definitive CRT + consolidation durvalumab	Proceeding with surgery risks incomplete resection without benefit (41). Transition to PACIFIC paradigm (5-yr OS 42.9% vs 33.4%, HR 0.72) (10, 11); note PACIFIC enrolled *de novo* unresectable disease. Early MDT reassessment critical. (Expert opinion)
Unresectable stage III (any N2/N3)	Anatomically unresectable; or MDT consensus against surgery	Definitive concurrent CRT → consolidation durvalumab	PACIFIC established consolidation durvalumab as SOC (10, 11). PACIFIC-2: concurrent IO+CRT no PFS benefit (HR 0.85, P = 0.247), reinforcing sequential integration (45). Real-world support: PACIFIC-R (44). (Evidence-based)
Frail patient (ECOG ≥2 or limited organ reserve)	Poor functional status; high perioperative morbidity risk; comorbidity burden	Definitive CRT ± consolidation IO (with caution); or symptom-directed/palliative management	Perioperative trials enrolled ECOG 0–1 (5–8); extrapolation lacks evidence. Adapt intensity to functional reserve and comorbidity with MDT input and structured geriatric/functional assessment (2, 42, 56). Consider attenuated regimens, hypofractionated RT, or symptom-directed care. (Expert opinion)

IO, immunotherapy; CRT, chemoradiotherapy; TKI, tyrosine kinase inhibitor; pCR, pathological complete response; EFS, event-free survival; OS, overall survival; DFS, disease-free survival; HR, hazard ratio; CI, confidence interval; MDT, multidisciplinary team; ECOG, Eastern Cooperative Oncology Group; R0/R1/R2, margin status; STS, Society of Thoracic Surgeons; SOC, standard of care; RT, radiotherapy.

This framework is a multidisciplinary decision-support tool and has not been prospectively validated as a unified treatment pathway. Evidence-based versus expert-opinion designations are indicated in the Rationale column. N2 subgroup data represent exploratory analyses and should be interpreted as hypothesis-generating. Clinical scenarios are not mutually exclusive; patients may present with overlapping features requiring individualized judgment.

The evidence-based/expert-opinion designations added to the Rationale column reflect Reviewer #1, Comment 1.1.

For patients with single-station N2a disease, limited mediastinal involvement, peripheral T1–T2 primaries, and favorable tumor biology, neoadjuvant chemoimmunotherapy followed by surgical resection represents an appropriate strategy when high probability of R0 resection is anticipated. In selected favorable scenarios, particularly in patients with oncogene-driven tumors for whom adjuvant targeted therapy is planned, upfront surgery followed by guideline-concordant adjuvant therapy remains reasonable.

For patients with multistation N2b disease, bulky mediastinal adenopathy (≥3 cm), T4 tumors invading critical mediastinal structures, or anatomically complex presentations, treatment intensification is typically required. Perioperative chemoimmunotherapy may be favored when systemic micrometastatic risk dominates, while neoadjuvant chemoradiotherapy may be more appropriate when anatomical complexity is the dominant barrier to margin-negative resection. For unresectable stage III disease, definitive concurrent chemoradiotherapy followed by consolidation durvalumab remains the established standard of care.

Patients demonstrating inadequate response—including radiographic progression, persistent bulky nodal disease, or insufficient metabolic response—should undergo prompt multidisciplinary reassessment, with transition to definitive chemoradiotherapy and consolidation immunotherapy favored over high-risk surgery when R0 resection is unlikely. For frail patients with poor functional status (ECOG ≥2) or limited cardiopulmonary reserve, treatment intensity should be adapted to functional status and comorbidity burden, informed by structured geriatric and functional assessment tools such as those referenced in the NCCN guidelines (56). Modified curative-intent chemoradiotherapy and consolidation immunotherapy or best supportive care may be appropriate alternatives, recognizing that pivotal perioperative trials enrolled predominantly ECOG 0–1 patients (5–8).

The proposed framework is designed as a pragmatic multidisciplinary decision-support tool, not as a prescriptive guideline. Its primary purpose is to facilitate structured risk stratification and transparent discussion among thoracic surgeons, medical oncologists, radiation oncologists, and diagnostic specialists, particularly in borderline scenarios where trial data alone do not mandate a single strategy.

Although grounded in phase III evidence, the framework has not been prospectively validated as a unified treatment pathway. Therefore, its application should be individualized according to local expertise, institutional resources, patient preferences, comorbidity burden, and shared decision-making.

## Discussion and future directions

Stage III NSCLC management has evolved substantially with high-level evidence supporting neoadjuvant and perioperative chemoimmunotherapy, shifting practice toward more dynamic, biology-informed decision-making (42). The framework proposed herein (**Figure 1**) integrates anatomical resectability, nodal burden, tumor biology, and treatment response to support individualized decision-making in an increasingly complex therapeutic landscape.

Despite consistent improvements in pathological response and EFS across perioperative trials, with OS benefit confirmed in selected settings, important uncertainties remain regarding optimal treatment intensity, sequencing, and duration. Current perioperative trial designs mandate continuation of adjuvant immunotherapy regardless of pathological response, yet hypothesis-generating exploratory analyses from conference presentations suggest that the incremental benefit of prolonged adjuvant therapy may be attenuated in patients achieving pCR (30). These findings raise clinically relevant questions regarding response-adapted strategies while underscoring the need for prospective validation before de-escalation can be safely implemented.

Emerging biomarkers, particularly pCR and ctDNA dynamics, offer promising tools for postoperative risk stratification and future personalization of perioperative therapy (30, 32–35). However, current evidence does not support routine escalation or de-escalation based on these biomarkers outside clinical trials, and their integration into decision-making should remain investigational. Similarly, perioperative chemoimmunotherapy demonstrates benefit across PD-L1 subgroups, reinforcing guideline recommendations against restricting treatment solely based on PD-L1 expression (56). Together, these data highlight the limitations of single static biomarkers and emphasize the need for integrated, multidimensional approaches that combine biological features, anatomical risk, nodal burden, and dynamic response assessment.

Despite these advances, substantive unmet needs persist. Across major perioperative trials, approximately 70–80% of patients do not achieve pCR, yet the recurrence patterns and underlying biology of these non-pCR tumors remain incompletely characterized (5–8, 30). This group represents a major population with persistent recurrence risk and should be prioritized for biomarker-driven intensification strategies, novel non-cross-resistant systemic approaches, and rational combinations targeting resistance pathways. Conversely, patients achieving deep pathological and molecular responses may ultimately be candidates for treatment de-escalation, but such strategies require prospective randomized evaluation before clinical implementation. Thus, the future challenge is not simply to add more therapy, but to identify which patients require intensification, which patients can safely avoid overtreatment, and which biomarkers can reliably support these decisions.

In parallel, broader representation of older, frail, and comorbid patients is needed, as cardiopulmonary reserve, interstitial lung disease, chronic obstructive pulmonary disease, and cardiovascular comorbidity may simultaneously affect surgical candidacy and immune-related toxicity risk. Standardized perioperative toxicity algorithms, patient-reported outcomes, cost-effectiveness analyses, and ctDNA workflow integration should be incorporated into future trial designs and real-world studies.

The next era of clinical investigation in stage III NSCLC should move beyond uniform one-size-fits-all perioperative regimens toward biologically adapted, response-stratified strategies. Specific testable hypotheses include: (i) ctDNA-guided treatment intensification in high-risk patients with persistent molecular residual disease, potentially incorporating novel systemic agents or consolidative radiotherapy; (ii) selective pCR-based de-escalation, particularly in patients achieving pCR with concurrent ctDNA clearance, through omission or shortening of adjuvant immunotherapy to mitigate cumulative toxicity, treatment burden, and financial burden; and (iii) prospective N2-enriched randomized trials directly comparing neoadjuvant-only versus full perioperative regimens, stratified by single-station versus multistation nodal disease or by clinically relevant biological and anatomical risk factors.

Ultimately, optimizing outcomes in stage III NSCLC will depend on flexible, individualized multidisciplinary approaches that integrate robust clinical trial evidence with emerging biological insights, local expertise, and patient-centered considerations.

## Conclusions

Neoadjuvant and perioperative chemoimmunotherapy have redefined the treatment landscape for resectable stage III NSCLC, supported by phase III data demonstrating improvements in pathological response and EFS, with OS benefit confirmed in key trials including CheckMate 816 and KEYNOTE-671. Optimal application, however, requires careful individualization within a multidisciplinary framework integrating anatomical complexity, nodal burden, tumor biology, and dynamic treatment response.

The risk-adaptive algorithm proposed herein (**Figure 1**) offers practical guidance across the heterogeneous spectrum of stage III disease, including selection between surgical and nonsurgical pathways, selection between neoadjuvant-only versus perioperative strategies, and management of inadequate response to induction therapy. Emerging biomarkers such as pCR and ctDNA hold promise for enhanced risk stratification and response-adaptive personalization, though prospective validation is required before routine clinical implementation.

Significant challenges remain, including defining optimal treatment duration and intensity, validating predictive and response-adaptive biomarkers, managing immune-related toxicities during the perioperative window, and addressing evidence gaps in underrepresented populations. Continued multidisciplinary collaboration, prospective clinical research, and translational biomarker integration will be essential to refine risk-adapted paradigms and improve outcomes for patients with stage III NSCLC.
